# Supplementary material for: Oral live attenuated polio vaccines induce enhanced T-cell responses with broad antigen recognition compared to inactivated polio vaccines
Source: medRxiv. 2025 May 21:2025.05.20.25328004. Preprint. [Version 1] doi: 10.1101/2025.05.20.25328004 (PMC12258767; doi:10.1101/2025.05.20.25328004)
Supplement: Supplement 2 [file media-2.pdf]

Supplementary Tables

Table S1: Demographics of volunteers enrolled in polio vaccine studies

Study Boosting (Vaccine type)	IPV-boosted Study (NCT04529538)			OPV-boosted Study (NCT03922061)				Combined Total
	fIPV only	fIPV + dmLT	Total	mOPV1	nOPV1	nOPV3	Total	
**Sample Size** n/N (%)								
Volunteers	10/29 (35.5%)	19/29 (65.5%)	29/57 (50.9%)	10/28 (35.7%)	16/28 (57.1%)	2/28 (7.1%)	28/57 (49.1%)	57/57 (100.0%)
**Priming History** n/N (%)								
IPV	10/10 (100.0%)	19/19 (100.0%)	29/29 (100.0%)	6/10 (60.0%)	4/16 (25.0%)	2/ 2 (100.0%)	12/28 (42.9%)	41/57 (72.0%)
OPV	–	–	–	4/10 (40.0%)	12/16 (75.0%)	–	16/28 (57.1%)	16/57 (28.0%)
**Ethnicity** n/N (%)								
Non-Hispanic	9/10 (90.0%)	17/19 (89.5%)	26/29 (89.7%)	10/10 (100.0%)	15/16 (93.8%)	2/ 2 (100.0%)	27/28 (96.4%)	53/57 (93.0%)
Hispanic	1/10 (10.0%)	2/19 (10.5%)	3/29 (10.3%)	0/10 (0.0%)	1/16 (6.3%)	0/ 2 (0.0%)	1/28 (3.6%)	4/57 (7.0%)
**Race** n/N (%)								
White	9/10 (90.0%)	19/19 (100.0%)	28/29 (96.6.%)	9/10 (90.0%)	16/16 (100.0%)	2/ 2 (100.0%)	27/28 (96.4%)	55/57 (96.5%)
Black/African American	1/10 (10.0%)	0/19 (0.0%)	1/29 (3.4%)	1/10 (10.0%)	0/16 (0.0%)	0/ 2 (0.0%)	1/28 (3.6%)	2/57 (3.5%)
**Sex** n/N (%)								
Male	1/10 (10.0%)	7/19 (36.8%)	8/29 (27.6%)	5/10 (50.0%)	7/16 (43.8%)	1/ 2 (50.0%)	13/28 (46.4%)	21/57 (36.8%)
Female	9/10 (90.0%)	12/19 (63.2%)	21/29 (72.4%)	5/10 (50.0%)	9/16 (56.3%)	1/ 2 (50.0%)	15/28 (53.6%)	36/57 (63.2%)
**Age** Years								
Mean (SD)	18.8 (0.632)	19.0 (0.667)	18.9 (0.651)	25.2 (7.52)	28.0 (5.56)	20 (1.41)	26.4 (6.42)	22.6 (5.86)
Median	19	19	19	22.5	27.5	20	25.5	20
Min, Max	18, 20	18, 20	18, 20	19, 40	20, 39	19, 21	19, 40	18, 40

IPV: inactivated poliovirus vaccine; OPV: oral poliovirus vaccine; fIPV: fractional dose trivalent inactivated poliovirus vaccine IPV; dmLT: LT(R192G/L211A; n: sample number; N: total samples; mOPV1: monovalent Sabin strain oral poliovirus vaccine serotype 1; nOPV1: monovalent novel oral poliovirus vaccine serotype 1; nOPV3: monovalent novel oral poliovirus vaccine serotype 3; SD: standard deviation; min: minimum; max: maximum.

Supplementary Tables

Table S2: Serotype specific serum neutralizing antibody (SNA) titers

Study	IPV-boosted Study (NCT04529538)			OPV-boosted Study (NCT03922061)				Combined Total
	fIPV only	fIPV + dmLT	Total	mOPV1	nOPV1	nOPV3	Total	
**Serotype-specific SNA titers**<br>Geometric Mean Titer (log_2)<br>(min. Titer, max. Titer)								
**PV1**								
Baseline	5.657<br>(2.50, 9.17)	4.446<br>(2.50, 10.50)	4.831<br>(2.50, 10.50)	10.248<br>(8.50, 10.50)	5.893<br>(2.50, 9.83)	8.333<br>(8.17, 8.50)	6.003<br>(2.50, 9.83)	5.364<br>(2.50, 10.50)
Day 28	9.739<br>(7.50, 10.50)	10.037<br>(6.83, 10.50)	9.934<br>(6.83, 10.50)	10.248<br>(8.50, 10.50)	10.306<br>(9.50, 10.50)	8.138<br>(7.50, 8.83)	10.113<br>(7.50, 10.50)	10.026<br>(6.83, 10.50)
**PV2**								
Baseline	5.719<br>(3.17, 10.50)	5.502<br>(3.17, 10.50)	5.576<br>(3.17, 10.50)	6.746<br>(2.83, 10.17)	6.126<br>(2.50, 10.50)	5.347<br>(3.50, 8.17)	6.263<br>(2.50, 10.50)	5.897<br>(2.50, 10.50)
Day 28	10.043<br>(9.17, 10.50)	10.072<br>(8.17, 10.50)	10.062<br>(8.17, 10.50)	8.721<br>(7.17, 10.50)	7.993<br>(2.50, 10.50)	5.191<br>(3.17, 8.50)	7.995<br>(2.50, 10.50)	8.970<br>(2.50, 10.50)
**PV3**								
Baseline	6.979<br>(2.50, 10.50)	6.856<br>(2.50, 10.50)	6.898<br>(2.50, 10.50)	6.313<br>(2.50, 10.50)	5.074<br>(2.50, 10.50)	7.576<br>(6.50, 8.83)	5.622<br>(2.50, 10.50)	6.250<br>(2.50, 10.50)
Day 28	10.343<br>(9.17, 10.50)	10.338<br>(9.50, 10.50)	10.340<br>(9.17, 10.50)	7.505<br>(2.50, 10.50)	6.218<br>(2.50, 10.50)	10.500<br>(10.50, 10.50)	6.904<br>(2.50, 10.50)	8.449<br>(2.50, 10.50)

IPV: inactivated poliovirus vaccine; OPV: oral poliovirus vaccine; fIPV: fractional dose trivalent inactivated poliovirus vaccine IPV; dmLT: LT(R192G/L211A; mOPV1: monovalent Sabin strain oral poliovirus vaccine serotype 1; nOPV1: monovalent novel oral poliovirus vaccine serotype 1; nOPV3: monovalent novel oral poliovirus vaccine serotype 3; PV1: poliovirus serotype 1; PV2: poliovirus serotype 2; PV3: poliovirus serotype 3; min: minimum; max: maximum.

Table S3: CD4+ and CD8+ T cell responses by day

Boosting (Study) (Vaccine type)	IPV-boosted Study (NCT04529538) (fIPV +/- dmLT)			OPV[a] -boosted Study (NCT03922061)			Total	Comp$_1$	Comp$_2$
**Priming History**	**IPV (N = 29)**	**OPV (N = 0)**	**IPV Study All (N = 29)**	**IPV (N = 12)**	**OPV (N = 16)**	**OPV Study All (N = 28)**	**(N = 57)**	**p-value q-value**	**p-value q-value**
**Number (n/N (%)) of volunteers with CD4+ T cell response by timepoint**									
Baseline	3 (10.3%)	–	3 (10.3%)	2 (16.7%)	1 (6.3%)	3 (10.7%)	6 (10.5%)	1.0000 1.0000	1.0000 1.0000
Day 7	0 (0.0%)	–	0 (0.0%)	5 (41.7%)	7 (43.8%)	12 (42.9%)	12 (21.1%)	0.7744 0.9152	**0.0005**
Day 10	3 (10.3%)	–	3 (10.3%)	–	–	–	3 (10.3%)*	–	–
Day 28	–	–	–	3 (25.0%)	9 (56.3%)	12 (42.9%)	12 (42.9%)	0.1460 0.4195	–
All	6 (20.7%)	–	6 (20.7%)	5 (41.7%)	11 (68.8%)	16 (57.1%)	22 (38.6%)	0.2101 0.4419	0.0525 0.2275
**Mean[b] (min, max) frequency (% parent) of CD4+ T cell response by timepoint**									
Baseline	0.073 (0.026, 0.144)	–	0.073 (0.026, 0.144)	0.034 (0.024, 0.045)	0.023 (0.023, 0.023)	0.031 (0.023, 0.045)	0.052 (0.023, 0.144)	–	0.3607 0.6116
Day 7	0.000 (0.000, 0.000)	–	0.000 (0.000, 0.000)	0.092 (0.025, 0.315)	0.059 (0.023, 0.123)	0.073 (0.023, 0.315)	0.073 (0.022, 0.315)	0.5966 0.7506	0.0161 0.0897
Day 10	0.037 (0.024, 0.059)	–	0.037 (0.024, 0.059)	–	–	–	0.037 (0.024, 0.059)	–	–
Day 28	–	–	–	0.112 (0.063,0.208)	0.074 (0.023, 0.211)	0.083 (0.023, 0.211)	0.083 (0.023, 0.211)	0.5157 0.6968	–
All	0.055 (0.024, 0.144)	–	0.055 (0.024, 0.144)	0.087 (0.024, 0.315)	0.065 (0.023, 0.211)	0.073 (0.023, 0.315)	0.070 (0.023, 0.315)	0.5181 0.6968	0.4555 0.6707
**Number (n/N (%)) of volunteers with CD8+ T cell response by timepoint**									
Baseline	8 (27.6%)	–	8 (27.6%)	2 (16.7%)	1 (6.3%)	3 (10.7%)	11 (19.3%)	1.0000 1.0000	0.2266 0.4419
Day 7	4 (13.8%)	–	4 (13.8%)	2 (16.7%)	3 (18.8%)	5 (17.9%)	9 (15.8%)	1.0000 1.0000	0.2668 0.4730
Day 10	3 (10.3%)	–	3 (10.3%)	–	–	–	3 (10.3%)*	–	–
Day 28	–	–	–	2 (16.7%)	3 (18.8%)	5 (17.9%)	5 (17.9%)*	1.0000 1.0000	–
All	11 (37.9%)	–	11 (37.9%)	3 (25.0%)	5 (31.3%)	8 (28.6%)	19 (33.3%)	0.7266 0.8855	0.2005 0.4419
**Mean[b] (min, max) frequency of CD8+ T cell response by timepoint**									
Baseline	0.071 (0.039, 0.174)	–	0.071 (0.039, 0.174)	0.079 (0.037, 0.121)	0.056 (0.056, 0.056)	0.071 (0.037, 0.121)	0.071 (0.037, 0.174)	–	0.9799 1.0000
Day 7	0.068 (0.033, 0.082)	–	0.068 (0.033, 0.082)	0.301 (0.211, 0.391)	0.067 (0.035, 0.090)	0.161 (0.035, 0.391)	0.119 (0.033, 0.391)	0.2234 0.4419	0.2255 0.4419
Day 10	0.046 (0.033, 0.054)	–	0.046 (0.033, 0.054)	–	–	–	0.046 (0.033, 0.054)	–	–
Day 28	–	–	–	0.168 (0.097, 0.238)	0.070 (0.037, 0.093)	0.109 (0.037, 0.238)	0.109 (0.037, 0.238)	–	–
All	0.065 (0.033, 0.174)	–	0.065 (0.033, 0.174)	0.183 (0.037, 0.391)	0.067 (0.035, 0.093)	0.120 (0.035, 0.391)	0.091 (0.033, 0.391)	0.0740 0.2886	0.0856 0.3035

IPV: inactivated poliovirus vaccine; OPV: oral poliovirus vaccine; fIPV: fractional dose trivalent inactivated poliovirus vaccine IPV; dmLT: LT(R192G/L211A;n: sample number; N: total samples; min: minimum; max: maximum.

[a] Inclusive of all OPV vaccine variations (monovalent and novel; PV1 and PV3)

[b] Means and proportions inclusive only of samples with CD4+ reactivity detected

[c] Frequency = summed background subtracted pct. of parent only for samples meeting response criteria

"_" denotes no samples collected: No day 28 for Study #1, no OPV Primed in Study #1, No day 10 for Study #2

Comp$_1$ is comparison of IPV vs OPV Priming within the OPV-boosted study

Comp$_2$ is comparison of IPV vs OPV Boosting across studies

Supplementary Tables

Table S4: Proportion of samples with CD4+ and CD8+ T cells reactive to structural, non-structural, or both peptide megapools

	Baseline	Day 7	Day 10	Day 28	Total
**Number (n / N (%))** of samples with CD4+ T cells reactive to Structural peptide megapool alone (Prime/Boost)					
IPV/IPV	1/29 (3.4%)	0/29 (0.0%)	2/29 (6.9%)	_	3/ 87 (3.5%)
IPV/OPV	1/12 (8.3%)	2/12 (16.7%)	_	1/12 (8.3%)	4/ 36 (11.1%)
OPV/OPV	0/16 (0.0%)	1/16 (6.2%)	_	1/16 (6.2%)	2/ 48 (4.2%)
All	2/57 (3.5%)	3/57 (5.3%)	2/29 (6.9%)	2/28 (7.1%)	9/171 (5.3%)
**Number (n / N (%))** of samples with CD4+ T cells reactive to Nonstructural peptide megapool alone					
IPV/IPV	1/29 (3.4%)	0/29 (0.0%)	0/29 (0.0%)	_	1/ 87 (1.2%)
IPV/OPV	1/12 (8.3%)	2/12 (16.7%)	_	0/12 (0.0%)	3/ 36 (8.3%)
OPV/OPV	1/16 (6.2%)	3/16 (18.8%)	_	4/16 (25.0%)	8/ 48 (16.7%)
All	3/57 (5.3%)	5/57 (8.8%)	0/29 (0.0%)	4/28 (14.3%)	12/171 (7.0%)
**Number (n / N (%))** of samples with CD4+ T cells reactive to both Structural and Non-structural peptide megapools					
IPV/IPV	1/29 (3.4%)	0/29 (0.0%)	1/29 (3.4%)	_	2/ 87 (2.3%)
IPV/OPV	0/12 (0.0%)	1/12 (8.3%)	_	2/12 (16.7%)	3/ 36 (8.3%)
OPV/OPV	0/16 (0.0%)	3/16 (18.8%)	_	4/16 (25.0%)	7/ 48 (9.0%)
All	1/57 (1.8%)	4/57 (7.0%)	1/29 (3.4%)	6/28 (21.4%)	12/171 (7.0%)
**Total**	6/57 (10.5%)	12/57 (21.1%)	3/29 (10.3%)	12/28 (42.9%)	33/171 (19.3%)
**Number (n / N (%))** of samples with CD8+ T cells reactive to Structural peptide megapool alone					
IPV/IPV	2/29 (6.9%)	1/29 (3.4%)	3/29 (10.3%)	_	6/ 87 (6.9%)
IPV/OPV	2/12 (16.7%)	1/12 (8.3%)	_	0/12 (0.0%)	3/ 36 (8.3%)
OPV/OPV	1/16 (6.2%)	0/16 (0.0%)	_	0/16 (0.0%)	1/ 48 (2.1%)
All	5/57 (8.8%)	2/57 (3.5%)	3/29 (10.3%)	0/28 (0.0%)	10/171 (5.9%)
**Number (n / N (%))** of samples with CD8+ T cells reactive to Nonstructural peptide megapool alone					
IPV/IPV	6/29 (20.7%)	1/29 (3.4%)	0/29 (0.0%)	_	7/ 87 (8.1%)
IPV/OPV	0/12 (0.0%)	0/12 (0.0%)	_	0/12 (0.0%)	0/ 36 (0.0%)
OPV/OPV	0/16 (0.0%)	3/16 (18.8%)	_	2/16 (12.5%)	5/ 48 (10.4%)
All	6/57 (10.5%)	4/57 (7.0%)	0/29 (0.0%)	2/28 (7.1%)	12/171 (7.0%)
**Number (n / N (%))** of samples with CD8+ T cells reactive to both Structural and Non-structural peptide megapools					
IPV/IPV	0/29 (0.0%)	2/29 (6.9%)	0/29 (0.0%)	_	2/ 87 (2.3%)
IPV/OPV	0/12 (0.0%)	1/12 (8.3%)	_	2/12 (16.7%)	3/ 36 (8.3%)
OPV/OPV	0/16 (0.0%)	0/16 (0.0%)	_	1/16 (6.2%)	1/ 48 (2.1%)
All	0/57 (0.0%)	3/57 (5.3%)	0/29 (0.0%)	3/28 (10.7%)	6/171 (3.5%)
**Total**	11/57 (19.3%)	9/57 (15.8%)	3/29 (10.3%)	5/28 (17.9%)	28/171 (16.4%)

IPV: inactivated poliovirus vaccine; OPV: oral poliovirus vaccine; n: sample number; N: total samples.

Supplementary Tables

Table S5: Mean frequency of antigen-specific CD4+ and CD8+ T cells detected in samples reactive to structural, non-structural, or both peptide megapools

**Mean (Min, Max)** frequency of ag-specific CD4+ T cells (% of CD4+ T cells) detected in samples reactive to Structural peptide megapool alone (n = 9 / 33)					
IPV/IPV	0.0495 (0.0495, 0.0495)	–	0.0258 (0.0242, 0.0274)	–	0.0337 (0.0242, 0.0495)
IPV/OPV	0.0237 (0.0237, 0.0237)	0.0406 (0.0367, 0.0444)	–	0.0632 (0.0632, 0.0632)	0.0420 (0.0237, 0.0632)
OPV/OPV	–	0.0288 (0.0288, 0.0288)	–	0.0254 (0.0254, 0.0254)	0.0271 (0.0254, 0.0288)
All	0.0366 (0.0237, 0.0495)	0.0367 (0.0288, 0.0444)	0.0258 (0.0242, 0.0274)	0.0443 (0.0254, 0.0632)	0.0360 (0.0237, 0.0632)
**Mean (Min, Max)** frequency of ag-specific CD4+ T cells (% of CD4+ T cells) detected in samples reactive to Non-structural peptide megapools alone (n = 12 / 33)					
IPV/IPV	0.0258 (0.0258, 0.0258)	–	–	–	0.0258 (0.0258, 0.0258)
IPV/OPV	0.0452 (0.0452, 0.0452)	0.0323 (0.0250, 0.0396)	–	–	0.0366 (0.0250, 0.0452
OPV/OPV	0.0231 (0.0231, 0.0231)	0.0343 (0.0225, 0.0449)	–	0.0462 (0.0231, 0.0675)	0.0389 (0.0225, 0.0675)
All	0.0314 (0.0231, 0.0452)	0.0335 (0.0225, 0.0449)	–	0.0462 (0.0231, 0.0675)	0.0372 (0.0225, 0.0675)
**Mean (Min, Max)** frequency of ag-specific CD4+ T cells (% of CD4+ T cells) detected in samples reactive to both Structural and Non-structural peptide megapools [1] (n = 12 / 33)					
IPV/IPV	0.1441 (0.1441, 0.1441)	–	0.0582 (0.0592, 0.0592)	–	0.1016 (0.0592, 0.1441)
IPV/OPV	–	0.3151 (0.3151, 0.3151)	–	0.1367 (0.0654, 0.2080)	0.1962 (0.0654, 0.3151)
OPV/OPV	–	0.0944 (0.0492, 0.1300)	–	0.1132 (0.0578, 0.2111)	0.1051 (0.0492, 0.2111)
All	0.1441 (0.1441, 0.1441)	0.1495 (0.0492, 0.3150)	0.0582 (0.0592, 0.0592)	0.1211 (0.0578, 0.2111)	0.1273 (0.0492, 0.3151)
**Mean proportion (%)[2]** of total CD4+ T cell response reactive to Non-structural peptides in samples with both Structural and Non-structural responses detected (n = 12 / 33)					
IPV/IPV	70.83%	_	35.55%	_	53.19%
IPV/OPV	_	67.30%	_	36.86%	47.01%
OPV/OPV	_	58.24%	_	56.87%	57.46%
All	70.83%	60.50%	35.55%	50.20%	54.13%
**Mean (Min, Max)** frequency of ag-specific CD8+ T cells (% of CD8+ T cells) detected in samples reactive to Structural peptide megapool alone (n = 10 / 28)					
IPV/IPV	0.0411 (0.0401, 0.0421)	0.0794 (0.0794, 0.0794)	0.0459 (0.0331, 0.0537)	–	0.0499 (0.0331, 0.0794)
IPV/OPV	0.0792 (0.0370, 0.1210)	0.2114 (0.2114, 0.2114)	–	–	0.1233 (0.0370, 0.2114)
OPV/OPV	0.0558 (0.0558, 0.0558)	–	–	–	0.0558 (0.0558, 0.0558)
All	0.0593 (0.0370, 0.1210)	0.1454 (0.0794, 0.2114)	0.0459 (0.0331, 0.0537)	–	0.0725 (0.0331, 0.2114)
**Mean (Min, Max)** frequency of ag-specific CD8+ T cells (% of CD8+ T cells) detected in samples reactive to Nonstructural peptide megapool alone (n = 12 / 28)					
IPV/IPV	0.0804 (0.0387, 0.1740)	0.0332 (0.0332, 0.0332)	–	–	0.0737 (0.0332, 0.1744)
IPV/OPV	–	–	–	–	–
OPV/OPV	–	0.0670 (0.0350, 0.0904)	–	0.0583 (0.0368, 0.0799)	0.0635 (0.0350, 0.0904)
All	0.0804 (0.0387, 0.1740)	0.0585 (0.0332, 0.0904)	–	0.0583 (0.0368, 0.0799)	0.0694 (0.0332, 0.1740)
**Mean (Min, Max)** frequency of ag-specific CD8+ T cells (% of CD8+ T cells) detected in samples reactive to both Structural and Non-structural peptide megapools[1] (n = 6 / 28)					
IPV/IPV	–	0.0789 (0.0763, 0.0815)	–	–	0.0789 (0.0763, 0.0815)

Supplementary Tables

IPV/OPV	_	0.3908 (0.3908, 0.3908)	_	0.1678 (0.0973, 0.2380)	0.2422 (0.0973, 0.3908)
OPV/OPV	_	_	_	0.0933 (0.0933, 0.0933)	0.0933 (0.0933, 0.0933)
All	_	0.1829 (0.0763, 0.3908)	_	0.1430 (0.0933, 0.2380)	0.1630 (0.0763, 0.3908)
**Mean proportion (%)**[2] of total CD8+ T cell response reactive to Non-structural peptides in samples with both Structural and Non-structural responses detected					
IPV/IPV	_	53.05%	_	_	53.05%
IPV/OPV	_	32.86%	_	36.62%	32.70%
OPV/OPV	_	_	_	42.13%	42.13%
All	_	46.32%	_	35.79%	41.05%

IPV: inactivated poliovirus vaccine; OPV: oral poliovirus vaccine; ag: antigen; n: sample number; min: minimum; max: maximum.

[1] Summed Back. Sub. Mean (min., max.) is used for samples responding to both Structural and Non-Structural
[2] Mean(Non-Structural / Summed Back. Sub.) for samples responding to both Structural and Non-Structural
"_" denotes no samples collected: No day 28 for Study #1, no OPV Primed in Study #1, No day 10 for Study #2

Supplementary Tables

Table S6: Absolute cell counts calculated for samples with CD4+ and CD8+ T cell responses

OPV-boosted Study (NCT03922061)						
**Priming/Boosting**	**Mean Abs. Lymph. per mcL**	**Mean CD4+ T-Cell Pct. Lymph.**	**Mean Abs. CD4+ T-Cells per mcL**	**Mean CD4+ Ag(+) T cells Pct. Parent**	**Mean Abs. CD4+ Ag(+) T cells per mcL**	**Expected Abs. CD4+ Ag(+) T cells per 8 mL**
**Day 0**						
IPV Primed/OPV Boosted (n = 2 /12)	2265	44.37% (37.245, 51.485)	990 (920, 1061)	0.03% (0.024, 0.045)	0.34 (0.20, 0.52)	2693 (1600, 4160)
OPV Primed/OPV Boosted (n = 1/16)	1550	35.09% (35.094, 35.094)	544 (544, 544)	0.02% (0.023, 0.023)	0.13 (0.13, 0.13)	1001 (1001, 1001)
**All (n = 3/28)**	2027	41.28% (35.094, 51.485)	841 (544, 1061)	0.03% (0.023, 0.045)	0.26 (0.16, 0.47)	2086 (1280, 3760)
**Day 7**						
IPV Primed/OPV Boosted (n = 5/12)	2132	48.84% (34.529, 57.702)	1059 (656, 1575)	0.09% (0.025, 0.315)	0.97 (0.18, 3.88)	7794 (1440, 31040)
OPV Primed/OPV Boosted (n = 7/16)	1736	47.53% (36.345, 63.267)	808 (428, 1097)	0.06% (0.023, 0.13)	0.48 (0.15, 1.43)	3814 (1200, 11440)
**All (n = 12/28)**	1901	48.08% (34.529, 63.267)	913 (428, 1575)	0.07% (0.023, 0.315)	0.67 (0.15, 3.79)	5332 (1200, 30320)
**Day 28**						
IPV Primed/OPV Boosted (n = 3/12)	2077	52.49% (49.492, 54.469)	1094 (728, 1585)	0.11% (0.063, 0.208)	1.23 (0.65, 2.35)	9802 (5200, 18800)
OPV Primed/OPV Boosted (n = 9/16)	1624	45.60% (35.514, 65.884)	737 (504, 1021)	0.07% (0.023, 0.211)	0.55 (0.13, 2.26)	4363 (1040, 18080)
**All (n = 12/28)**	1738	47.33% (35.514, 65.884)	826 (504, 1585)	0.08% (0.023, 0.211)	0.69 (0.14, 2.42)	5485 (1120, 19360)
**All time-points**						
IPV Primed/OPV Boosted (n = 10/36)	2142	49.04% (34.529, 57.702)	1056 (656, 1585)	0.09% (0.024, 0.315)	0.92 (0.18, 3.89)	7350 (1440, 31120)
OPV Primed/OPV Boosted (n = 17/48)	1666	45.78% (35.094, 65.884)	755 (428, 1097)	0.07% (0.023, 0.211)	0.49 (0.13, 2.32)	3926 (1040, 18560)
**All (n = 27/84)**	1842	46.99% (34.529, 65.884)	866 (428, 1585)	0.07% (0.023, 0.315)	0.63 (0.15, 3.82)	5057 (1200, 30560)
**Priming/Boosting**	**Mean Abs. Lymph. per mcL**	**Mean CD8+ T-Cell Pct. Lymph.**	**Mean Abs. CD8+ T-Cells per mcL**	**Mean CD8+ Ag(+) T cells Pct. Parent**	**Mean Abs. CD8+ Ag(+) T cells per mcL**	**Expected Abs. CD8+ Ag(+) T cells per 8 mL**
**Day 0**						
IPV Primed/OPV Boosted (n = 2/12)	1875	15.25% (13.911, 16.593)	283 (280, 287)	0.08% (0.037, 0.121)	0.22 (0.10, 0.38)	1789 (800, 3040)
OPV Primed/OPV Boosted (n = 1/16)	1550	21.76% (21.762, 21.762)	337 (337, 337)	0.06% (0.056, 0.056)	0.19 (0.19, 0.19)	1510 (1510, 1510)
**All (n = 3/28)**	1767	17.42% (13.911, 21.762)	301 (280, 337)	0.07% (0.037, 0.121)	0.21 (0.09, 0.47)	1710 (720, 3760)
**Day 7**						
IPV Primed/OPV Boosted (n = 2/12)	1895	15.93% (15.56, 16.297)	302 (294, 310)	0.30% (0.211, 0.391)	0.91 (0.62, 1.21)	7272 (4960, 9680)
OPV Primed/OPV Boosted (n = 3/16)	1543	20.03% (16.318, 22.503)	320 (162, 468)	0.07% (0.035, 0.09)	0.21 (0.09, 0.31)	1715 (720, 2480)
**All (n = 5/28)**	1684	18.39% (15.56, 22.503)	313 (162, 468)	0.16% (0.035, 0.391)	0.50 (0.09, 1.48)	4031 (720, 11840)
**Day 28**						
IPV Primed/OPV Boosted (n = 2/12)	1660	17.43% (15.632, 19.223)	284 (261, 306)	0.17% (0.097, 0.238)	0.48 (0.25, 0.76)	3817 (2000, 6080)
OPV Primed/OPV Boosted (n = 3/16)	1607	18.08% (14.794, 20.481)	292 (225, 358)	0.07% (0.037, 0.093)	0.20 (0.09, 0.31)	1635 (720, 2480)
**All (n = 5/28)**	1628	17.82% (14.794, 20.481)	289 (225, 358)	0.11% (0.037, 0.238)	0.32 (0.09, 0.79)	2520 (720, 6320)
**All time-points**						
IPV Primed/OPV Boosted (n = 6/36)	1810	16.20% (13.911, 19.223)	290 (261, 310)	0.18% (0.037, 0.391)	0.53 (0.09, 1.36)	4246 (720, 10880)
OPV Primed/OPV Boosted (n = 7/48)	1571	19.44% (14.794, 22.503)	311 (162, 468)	0.07% (0.035, 0.093)	0.21 (0.08, 0.33)	1667 (640, 2640)
**All (n = 13/84)**	1682	17.95% (13.911, 22.503)	301 (162, 468)	0.12% (0.035, 0.391)	0.36 (0.08, 1.48)	2890 (640, 11840)

Supplementary Tables

mcL: microliter; Abs: absolute; lymph: lymphocyte; pct: percent; ag: antigen; IPV: inactivated poliovirus vaccine; OPV: oral poliovirus vaccine; n: sample number.

Calculations only include samples that met response criteria; N will therefore be different at each timepoint

Supplementary Tables

Table S7: Proportion of volunteers with fecal poliovirus shedding detected by day

OPV-boosted Study (NCT03922061)				
**Priming History**	**Proportion (n/N) study participants with PCR-positive fecal sample**			
	**Day 7**	**Day 14**	**Day 21**	**Day 28**
IPV-primed	12/12 (100.0 %)	10/12 (83.3%)	5/12 (41.7%)	4/12 (33.3%)
OPV-primed	11/16 (68.8%)	7/16 (43.8%)	5/16 (31.3%)	0/16 (0.0%)
All	23/28 (82.1%)	17/28 (60.7%)	10/28 (35.7%)	4/28 (14.3%)
	**Mean log_{10} $CCID_{50}$ viral titer**			
	**Day 7**	**Day 14**	**Day 21**	**Day 28**
IPV-primed	4.24 (3.83, 4.69)	2.92 (2.71, 3.15)	2.85 (2.57, 3.17)	2.95 (2.38, 3.67)
OPV-primed	3.15 (2.9, 3.41)	2.77 (2.72, 2.83)	2.75 (2.75, 2.75)	0.00 (0.00, 0.00)
All	3.67 (3.36, 4.02)	2.86 (2.74, 2.99)	2.80 (2.69, 2.92)	2.95 (2.38, 3.67)

IPV: inactivated poliovirus vaccine; OPV: oral poliovirus vaccine; n: sample number; N: total samples

[a] Inclusive of all volunteers enrolled in Study #2 and all OPV vaccine variations (monovalent and novel; PV1 and PV3).

[b] The titer value provided matches the vaccine serotype (poliovirus type 1 or 3) received by the volunteer. We considered any poliovirus type 1 or 3-specific stool neutralization titer >2 to be detectable.

Supplementary Tables

Table S8: Time to Shedding Cessation and Shedding Index Endpoint by priming cohort

OPV-boosted Study (NCT03922061)				
Boosting (Vaccine type)	OPV[a]			Comp$_5$
Priming History	IPV (n = 12)	OPV (n = 16)	All (n = 28)	p-value q-value
**Time to shedding cessation[b] (Days)**				
Mean (95% CI)	26.833 (20.040, 33.627)	14.875 (8.652, 21.098)	20.000 (15.116, 24.884)	**3.475×10^{-11} 3.823×10^{-10}**
min	14	0	0	
max	42	28	42	
**Shedding Index Endpoint[c] (SIE)**				
Mean (95% CI)	2.229 (1.627, 2.831)	1.063 (0.557, 1.568)	1.563 (1.136, 1.989)	**0.0004 0.0039**
min	0.719	0.000	0.000	
max	2.305	3.625	3.625	

IPV: inactivated poliovirus vaccine; OPV: oral poliovirus vaccine; n: sample number; SIE: shedding index endpoint; CI: confidence interval; min: minimum; max: maximum.

[a] Inclusive of all OPV vaccine variations (monovalent and novel; PV1 and PV3).

[b] Time to shedding cessation was defined as the collection date of the first stool samples that tested negative for the presence of poliovirus by PCR. Shedding cessation was confirmed by two stool samples collected at least 24 hours apart that tested negative by PCR.

[c] SIE is calculated using $\log_{10}$ $CCID_{50}$ titer results from shared stool collection timepoints (Days 7, 14, 21, 28)

Comp$_5$ is comparison of IPV vs OPV Priming within Study #2

Comp$_5$ is calculated using Welch two-sample t test for difference of means.

Table S9: Time to Shedding Cessation and Shedding Index Endpoint by T cell response

OPV [d] -boosted Study (NCT03922061)						
**Boosting (Vaccine type)**	**OPV [d]**				**Comp$_6$**<br>**p-value**<br>**q-value**	**Comp$_7$**<br>**p-value**<br>**q-value**
**Priming History**	**IPV**		**OPV**			
**T cell response[a]**	**(n = 5)**<br>**(+)**	**(n = 7)**<br>**(–)**	**(n = 12)**<br>**(+)**	**(n = 4)**<br>**(–)**		
**Time to shedding cessation [b] (Days)**						
Mean<br>(95% CI)	29.40<br>(15.118, 43.682)	25.00<br>(15.212, 34.788)	17.50<br>(10.034, 24.966)	7<br>(-5.862, 19.862)	0.0772<br>0.1545	**<0.00005**<br>**<0.00005**
min	21	14	0	0		
max	42	42	21	14		
**Shedding Index Endpoint (SIE)[c]**						
Mean<br>(95% CI)	2.52<br>(1.44, 3.59)	2.02<br>(1.09, 2.96)	1.30<br>(0.69, 1.91)	0.35<br>(-0.29, 0.98)	0.3895<br>0.6329	**0.0156**<br>**0.0897**
min	1.77	0.72	0.00	0.00		
max	3.63	3.25	2.30	0.70		

IPV: inactivated poliovirus vaccine; OPV: oral poliovirus vaccine; n: sample number; SIE: shedding index endpoint; CI: confidence interval; min: minimum; max: maximum.

[a] T cell response is inclusive of detection of CD4+ and/or CD8+ T cell reactivity at any timepoint that met response criteria

[b] Time to shedding cessation was defined as the collection date of the first stool samples that tested negative for the presence of poliovirus by PCR. Shedding cessation was confirmed by two stool samples collected at least 24 hours apart that tested negative by PCR.

[c] SIE is calculated using $\log_{10}$ $CCID_{50}$ titer results from shared stool collection timepoints (Days 7, 14, 21, 28)

[d] Inclusive of all OPV vaccine variations (monovalent and novel; PV1 and PV3).

Comp$_6$ is comparison of T cell (+) vs T cell (–) within IPV Primed cohort

Comp$_7$ is comparison of T cell (+) vs T cell (–) within OPV Primed cohort

Comparisons calculated using Welch two-sample t test for difference of means.
