# Supplementary material for: Oral live attenuated polio vaccines induce enhanced T-cell responses with broad antigen recognition compared to inactivated polio vaccines
Source: medRxiv. 2025 May 21:2025.05.20.25328004. Preprint. [Version 1] doi: 10.1101/2025.05.20.25328004 (PMC12258767; doi:10.1101/2025.05.20.25328004)
Supplement: 3 [file NIHPP2025.05.20.25328004v1-supplement-3.pdf]

medRxiv preprint doi: https://doi.org/10.1101/2025.05.20.25328004; this version posted May 21, 2025. The copyright holder for this preprint (which was not certified by peer review) is the author/funder, who has granted medRxiv a license to display the preprint in perpetuity.
It is made available under a CC-BY 4.0 International license .

Supplemental Methods

Clinical trial details

Samples and data from human subjects included in the current study originated from participants enrolled in polio vaccine studies conducted at the University of Vermont Vaccine Testing Center (Burlington, VT (USA)). The first study (#NCT04529538) was completed in 2019 and complete trial results are previously reported(19). The study protocol was approved by the United State Food and Drug Administration's (FDA) Investigational New Drug program (IND#18511) and by the Institutional Review Board (IRB) at the University of Vermont (UVM). This study was conducted in compliance with the ethical principles of the Declaration of Helsinki and all participants provided written, informed consent for use of their data and samples in future research.

medRxiv preprint doi: https://doi.org/10.1101/2025.05.20.25328004; this version posted May 21, 2025. The copyright holder for this preprint (which was not certified by peer review) is the author/funder, who has granted medRxiv a license to display the preprint in perpetuity.
It is made available under a CC-BY 4.0 International license .

The second multisite study (#NCT03922061) was completed in 2023, and complete results will be reported separately (manuscript currently under preparation). The study protocol for study #NCT03922061 was approved by the United State Food and Drug Administration's (FDA) Investigational New Drug program (IND# 026305) Advarrra IRB and the University of Vermont (UVM) IRB. This study was conducted in compliance with the ethical principles of the Declaration of Helsinki and all participants provided written, informed consent. Participants enrolled at UVM were invited to participate in a separate substudy (STUDY0001320) to provide additional blood samples for the work presented here. Volunteers who agreed to substudy participation provided written, informed consent for use of their data and samples in future research.

Testing of serum and stool samples for both studies was reviewed by CDC, deemed research not involving human subjects, and was conducted consistent with applicable federal law and CDC policy (See e.g., 45 C.F.R. part 46; 21 C.F.R. part 56; 42 U.S.C. §241(d), 5 U.S.C. §552a, 44 U.S.C. §3501 et seq.)

Serum neutralizing antibody titers

For both studies, (NCT04529538; NCT03922061), serum samples were collected for vaccine humoral immunogenicity assessments. For participants enrolled in study #NCT04529538, serum samples were collected on Day 0 prior to dosing, and on Days 7, 10, 14, and 28. For study #NCT03922061 serum samples were collected at the screening visit and on Day 28 for all cohorts. All samples were aliquoted and stored at ≤-20°C before being shipped to Polio and Picornavirus Branch at the United States Centers for Disease Control and Prevention (CDC) for assessment of type-specific polio neutralizing antibody titers(47). To enable comparison

medRxiv preprint doi: https://doi.org/10.1101/2025.05.20.25328004; this version posted May 21, 2025. The copyright holder for this preprint (which was not certified by peer review) is the author/funder, who has granted medRxiv a license to display the preprint in perpetuity.
It is made available under a CC-BY 4.0 International license .

across studies, only results from baseline and day 28 samples are included in the current analysis.

Poliovirus shedding

For study (NCT03922061), stool samples were collected on Days 7, 14, 21, 28 for all volunteers and additionally on Days 2, 4, and 9 for IPV-primed cohorts to detect and assess the quantity of virus shed and to confirm cessation of shedding. Samples were processed at the University of Vermont and stored at ≤-20°C before being transported to the Polio and Picornavirus Branch at the United States Centers for Disease Control and Prevention (CDC). The detection of type-specific poliovirus in stool was determined via multiplex real-time polymerase chain reaction (PCR). In samples positive by PCR, type-specific infectious virus was quantified as the 50% cell culture infectious dose ($CCID_{50}$) per gram of stool.

The mean viral RNA titers and Shedding Index Endpoints (SIE) were calculated as the arithmetic mean across each group. The common time points (Days 7, 14, 21, and 28 post-vaccination) were used in the calculation of the mean SIE, which included zeroes for PCR negative observations. The arithmetic mean of time to shedding cessation (days) across each group were compared as opposed to a time-to-event analysis as the data were not censored; stool samples were continually collected until cessation of viral shedding was observed for each subject.

Identification of Poliovirus-Specific T cell Reponses by Flow Cytometry

Design of Polio Peptide Pools

medRxiv preprint doi: https://doi.org/10.1101/2025.05.20.25328004; this version posted May 21, 2025. The copyright holder for this preprint (which was not certified by peer review) is the author/funder, who has granted medRxiv a license to display the preprint in perpetuity.
It is made available under a CC-BY 4.0 International license .

To develop a pool of representative poliovirus peptides for research into poliovirus specific lymphocytes, polyprotein sequences available in the Virus Pathogen Database and Analysis Resource (ViPR) were analyzed for sequence redundancy. Nine strains available in ViPR were used in this analysis: two human poliovirus 1 strains Mahoney (GenBank Accessions: V01149, V01148), one human poliovirus 1 strain Mahoney_CDC (GenBank Accession: KU866422), three human poliovirus 1 strains Sabin 1 (GenBank Accessions: AY184219, V01150, GQ984141), one human poliovirus 2 strain MEF-1 (GenBank Accession: AY238473), one human poliovirus 2 strain Sabin 2 (GenBank Accession: AY184220), and one human poliovirus 3 strain Sabin 3 (GenBank Accession: AY184221). Per each sequence 15-mers overlapping by 10 amino acids were generated using the cluster tools used with 70% conservancy (PMID: 30014462) available in IEDB (PMID: 31114900). As a result, we identified a total of 661 peptides, 134 were uniquely represented in either of the strains while 527 were represented in one or more of the representative strains. The 661 peptides were synthetized by A&A (San Diego) as crude material. Peptide assignment to each antigen was performed as previously reported taking into account mapping positions across several Enteroviruses isolates including Polioviruses(48).

Peptides were resuspended in DMSO, pooled as structural (STR; n = 293) or nonstructural (NON; n = 368). To generate the two different megapools, the peptide pools followed a sequential lyophilization approach previously published(49). The resulting lyocake was resuspended in DMSO at a stock concentration of 1mg/mL generating the STR and NON megapools. As a control, a SARS-CoV-2 spike megapool (COV) was also utilized based on 15-mers overlapping by 10 amino acids spanning the spike SARS-CoV-2 ancestral strain (GenBank: MN_908947)(50). Megapools were generated by the Grifoni and Sette Labs at La

medRxiv preprint doi: https://doi.org/10.1101/2025.05.20.25328004; this version posted May 21, 2025. The copyright holder for this preprint (which was not certified by peer review) is the author/funder, who has granted medRxiv a license to display the preprint in perpetuity. It is made available under a CC-BY 4.0 International license .

Jolla Institute for Immunology and were maintained at -20 degrees Celsius with a maximum of one thaw prior to use in this assay.

Isolation of Peripheral Blood Mononuclear Cells (PBMCs)

PBMCs were isolated by University of Vermont Vaccine Testing Center personnel according to standard procedures. Briefly, whole blood was collected from study participants in EDTA tubes and layered onto Histopaque-1077 (Sigma #H8889) for centrifugation. PBMCs were resuspended in cell freezing media (Sigma #C6164) and 6-10 million PBMCs were aliquoted per cryovial. Cryovials were frozen down in Mr. Frosty containers at -80 degrees Celsius and then transferred to liquid nitrogen for long-term storage.

Thawing of Peripheral Blood Mononuclear Cells (PBMCs)

RPMI-1640 (Cytiva #SH30096.02) supplemented with heat-inactivated fetal bovine serum ("FBS", Corning #35-011-CV, 10%), L-Glutamine (Gibco #A2916802, 1%), and Penicillin-Streptomycin (Gibco #15070063, 1%), was used as a base media throughout this protocol and is referred to as "RPMI-C." Cryovials were removed from liquid nitrogen and placed in a water bath at 37 degrees Celsius to thaw for 60 seconds. One mL of sterile RPMI-C containing Benzonase (Sigma-Aldrich #E1014-25KU, working concentration 50 units/mL) was added to each cryovial and contents were briskly poured into conical tubes containing 3 mL sterile RPMI-C. Conical tubes were centrifuged at 1200 rpm for 10 minutes at 10 degrees Celsius and the supernatant was removed by gentle pouring. Cell pellets were resuspended in 1 mL RPMI-C and centrifuged at the same settings. The supernatant was removed by aspiration and cell pellets were resuspended in 1 mL RPMI-C and pipetted through 70 µM filters (PluriSelect #43-

medRxiv preprint doi: https://doi.org/10.1101/2025.05.20.25328004; this version posted May 21, 2025. The copyright holder for this preprint (which was not certified by peer review) is the author/funder, who has granted medRxiv a license to display the preprint in perpetuity.
It is made available under a CC-BY 4.0 International license .

10070-70). Filtered PBMCs proceeded immediately to staining for the unstimulated panel or to overnight stimulation for the AIM panel.

Cell Surface Staining for Detection of Activation Induced Markers (AIM)

Activation induced cell marker (AIM) expression was assessed by flow cytometry as previously described(50). All timepoints and experimental conditions ((PHA positive control, DMSO negative controls, stimulation with non-structural (NON) peptide pool, stimulation with structural (STR) peptide pool)) for each subject were included in the same experimental run.

Filtered PBMCs were seeded 50 µL per well in 96-well U-bottom plates followed by the addition of 4X treatments. Each clinical sample was stimulated with the following treatments: RPMI-C only control ("UNS"), phytohemagglutinin ("PHA", Roche #11249738001, 10.0 µg/mL) as a positive control, polio structural megapool ("STR",1.0 µg/mL), polio non-structural megapool ("NON", 1.0 µg/mL), and triplicate wells of dimethyl sulfoxide ("DMSO", Sigma #D8418) equimolar to the peptide megapools as a negative vehicle control. In addition to the polio megapools, a SARS-CoV-2 spike megapool ("COV", 1.0 µg/mL) was used to stimulate the donor control. The donor control samples were PBMCs from a single blood draw on one individual that were aliquoted into many cryovials, and one cryovial was included in each round of stimulation, staining, and sample acquisition. To stimulate cells for single-color controls, PBMCs were plated from a single individual with the same treatments described above. Using cells from a single individual allowed cells to be pooled following stimulation and then divided to ensure a mixture of activation states in each single-color control to capture the brightest protein expression for all markers. Plates were incubated 24 hours at 37 degrees Celsius and 5% CO_2. To generate a

medRxiv preprint doi: https://doi.org/10.1101/2025.05.20.25328004; this version posted May 21, 2025. The copyright holder for this preprint (which was not certified by peer review) is the author/funder, who has granted medRxiv a license to display the preprint in perpetuity.
It is made available under a CC-BY 4.0 International license .

sample specific unstained control for autofluorescence extraction ("unstained"), as well as the a4β7 AF700 fluorescence minus one ("FMO") control, 20 µL of cells were removed from the various treatment wells of a single sample, pooled, and plated into two wells for the respective staining protocols. Staining was performed in 96-well U-bottom plates for samples, donor control, FMO, and unstained controls and in in 1.5 mL Eppendorf tubes for single-color controls.

Following overnight stimulation, plates were centrifuged and supernatants discarded. Samples, donor control, and FMO were resuspended in 50 µL of UV Blue LiveDead (Invitrogen #L34962, 1:200), unstained was resuspended in 50 µL of phosphate buffered saline ("PBS", Corning #21-031-CV), and incubated at 4 degrees Celsius. To wash, 100 µL PBS 1% FBS was added to each well and plates were centrifuged and supernatants removed. The antibody cocktail was made in Brilliant Buffer (BD Horizon #566349) containing blocking antibodies (Fc Block, BioLegend #422302, 1:20; Monocyte Block, BioLegend #426103, 1:20) and all fluorescent antibodies (CD69 BUV661, BD #750213, 1:100; CD8 BUV805, BD #612890, 1:400; CD4 Pacific Blue, BioLegend #317424, 1:400; CD3 BV510, BioLegend #344828, 1:50; CD134/OX40 BV605, BioLegend #350028, 1:20; CD14 BV711, BioLegend #301838, 1:100; CD19 BV711, BioLegend #302246, 1:100; CXCR5 BV750, BioLegend #356942, 1:20; CD279/PD-1 PE, BioLegend #329906, 1:40; CD45 PerCP/Cy5.5, BioLegend #368504, 1:50; CD137 PE/Cy7, BioLegend #309818, 1:50; TCRgd APC, BioLegend #331212, 1:20) except a4β7AF700. Once 100 µL was set aside as the FMO cocktail, the final antibody was added (a4β7 AF700, R+D #FAB10078N, 1:40). Samples and donor control were resuspended in antibody cocktail, FMO in FMO cocktail, and unstained in PBS, plates were incubated at 4 degrees Celsius. Samples were washed in PBS 1% FBS, centrifuged and supernatants removed. For fixation, samples, donor control, FMO, and

medRxiv preprint doi: https://doi.org/10.1101/2025.05.20.25328004; this version posted May 21, 2025. The copyright holder for this preprint (which was not certified by peer review) is the author/funder, who has granted medRxiv a license to display the preprint in perpetuity.
It is made available under a CC-BY 4.0 International license .

unstained were resuspended in PBS 1% paraformaldehyde (Alfa Aesar #43386) and incubated at 4 degrees Celsius. A final wash of PBS 1% FBS was added to each well, plates were centrifuged, supernatants removed, samples resuspended in PBS, plates wrapped tightly with parafilm, and stored at 4 degrees Celsius. Immediately before acquisition, PBS was added to each well and samples were thoroughly mixed to resuspend cells.

The single-color control stains were prepared in 1.5 mL Eppendorf tubes at the dilutions indicated above, using PBS 1% FBS for all antibodies and the negative control, and PBS for UV Blue LIVE/DEAD. Following overnight stimulation, cells stimulated for the single-color controls were pooled in a reservoir. An aliquot of cells was removed from the reservoir into a tube and heated at 65 degrees Celsius for 10 minutes. An equal aliquot of cells was added to the tube of heat-killed cells, and the tube was centrifuged, supernatant aspirated and resuspended in the UV Blue LIVE/DEAD stain. The remaining cells in the reservoir were replated, centrifuged, and supernatants removed. Using 50 µL of the prepared stains, cell pellets were resuspended in the plate wells and transferred to tubes, incubated at 4 degrees Celsius. Samples were washed in PBS 1% FBS, centrifuged, and supernatant aspirated. Cell pellets were resuspended in 1% paraformaldehyde and incubated at 4 degrees Celsius. A final wash of PBS 1% FBS was added to each tube and centrifuged, and supernatant removed. Cells were resuspended in 400 µL PBS and transferred to capped 5 mL round bottom tubes for storage at 4 degrees Celsius.

Flow Cytometry Data Acquisition

Samples were acquired on the Aurora Cytek under settings set by passing daily QC, with the following adjustments: forward scatter 60, side scatter 220, threshold 300,000, forward area

medRxiv preprint doi: https://doi.org/10.1101/2025.05.20.25328004; this version posted May 21, 2025. The copyright holder for this preprint (which was not certified by peer review) is the author/funder, who has granted medRxiv a license to display the preprint in perpetuity.
It is made available under a CC-BY 4.0 International license .

scaling 1.0. Between 100,000-200,000 events were collected into the lymphocyte scatter gate for all single-color controls. To account for changes in autofluorescence over days of storage, the sample specific unstained control was acquired (25,000 events) each day that samples were acquired and applied to the live unmixing for those samples (only for AIM Panel). For the Unstimulated Panel, samples were run in tubes at a flow rate ~2,000 events/second, with a stop gate set to 500,000 events in the lymphocyte scatter gate. For the AIM Panel, samples were run in plates at a flow rate ~5-10,000 events/second, with stop gates set for time (2 minutes), volume (300 µL), and count (500,000 events into the lymphocyte scatter gate). Similar to the sample specific unstained control, the a4β7 AF700 FMO was acquired (25,000 events) each day that samples were acquired (only for AIM Panel).

Flow Cytometry Gating

The online cytometry analysis platform OMIQ (Dotmatics) was used for manual bidimensional gating of raw flow cytometry data. FCS files were uploaded to OMIQ into individual workflows corresponding to each experimental run. Scaling and gating were performed uniformly across all experimental runs. Scaling was first adjusted on all fluorescent features by using an Arcsinh transformation. A time gate was then individually adjusted for each FCS file, followed by application of a universally applied hierarchical gating scheme. PHA stimulated samples were scaled and gated separately. Additional scaling and gating for AIM+ (CD137+OX40+) CD4+ T cells and AIM+ (CD137+CD69+) CD8+ T cell gates was done at the plate level to account for experimental variation in fluorescence signal detection (see **Fig S1A**, gating scheme). Data, including cell counts and percent (%) parent gate, were then exported as .csv files and read into

medRxiv preprint doi: https://doi.org/10.1101/2025.05.20.25328004; this version posted May 21, 2025. The copyright holder for this preprint (which was not certified by peer review) is the author/funder, who has granted medRxiv a license to display the preprint in perpetuity.
It is made available under a CC-BY 4.0 International license .

R studio for downstream analysis (code available at https://github.com/P-Harvey/Ag_Spec_T_cell_PV).

Antigen-specific T cell Analysis

Following initial assay development in which the expression of various AIM makers were assessed after stimulation protocols in our laboratory, we down selected to those AIM markers with consistent and robust expression readily detectible in combination with our complete fluorophore antibody panel. For CD4+ T cells, CD137+OX40+ was chosen, and for CD8+ T cells CD137+CD69+ was chosen. The frequency of poliovirus-specific AIM+ (CD137+OX40+CD4+) and (CD137+CD69+CD8+) T cells was separately determined for each peptide stimulation condition (structural and non-structural peptide pools) using a background subtraction (BS) method, as previously reported(20). Briefly, the frequency (%) of AIM+ T cells was calculated by subtracting the percent (%) AIM+ cells detected in sample-specific DMSO negative vehicle controls (run in triplicate and averaged) from the percent (%) of AIM+ cells detected for each sample and AIM+ parameter combination assessed. If the averaged DMSO value was < LOS, the parameter-specific LOS value was used.

The assay limit of detection (LOD) was calculated for each AIM parameter by taking twice the upper bound of the 95% CI of the geometric mean of the arithmetic means ($\overline{DMSO}_{ik}$) of DMSO triplicates and taking twice the upper bound of the 95% CI. Any averaged DMSO values < 0.005 were imputed to 0.005. The LOD was calculated to be 0.0166% for AIM+CD4+ and 0.022% for AIM+CD8+ parameters.

medRxiv preprint doi: https://doi.org/10.1101/2025.05.20.25328004; this version posted May 21, 2025. The copyright holder for this preprint (which was not certified by peer review) is the author/funder, who has granted medRxiv a license to display the preprint in perpetuity.
It is made available under a CC-BY 4.0 International license .

The assay limit of sensitivity (LOS) was calculated for each AIM parameter as two standard deviations above the median of the averaged DMSO triplicates. The LOS was calculated to be 0.021% for AIM+CD4+ and 0.033% for AIM+CD8+ parameters.

The stimulation index (SI) was calculated for each sample and T cell/AIM+ parameter combination as the frequency (%) of AIM+ cells divided by the averaged DMSO values per parameter condition.

Samples with a BS value > LOD and > LOS, and with a SI > 2 met response criteria and were considered to represent a positive T cell response. AIM+ T cell frequencies for samples that did not meet positive response criteria were subsequently set to "0".

Total poliovirus-specific CD4+ and CD8+ T cells were calculated for samples that met response criteria by summing the frequency (%) of background subtracted AIM+ cells detected in structural and non-structural stimulation conditions per sample.

Calculation of Absolute Cell Counts

To estimate the absolute counts of Ag. specific T cells in whole blood, we first multiplied the absolute Lymphocyte count (provided by the UVMMC clinical laboratory for the corresponding paired peripheral blood sample (obtained at the same blood draw) by the Pct. (%) CD4+ (or CD8+) of Lymphocytes (as determined by flow cytometry). This value was then multiplied by the background subtracted percentage of parent (% CD4/CD137+OX40+ of CD4+ or CD8/CD137+CD69+ of CD8+) for each sample and treatment condition (stimulation with non-

medRxiv preprint doi: https://doi.org/10.1101/2025.05.20.25328004; this version posted May 21, 2025. The copyright holder for this preprint (which was not certified by peer review) is the author/funder, who has granted medRxiv a license to display the preprint in perpetuity.
It is made available under a CC-BY 4.0 International license .

structural or structural peptide pools), giving a treatment specific estimate of Ag. specific T cells per sample (cells/mcL). If a LAN responded to both treatments (non-structural and structural), the mean of the two treatments' cell counts were used in calculating the values in Table S6; if a sample responded to only one treatment, then those exact cell counts were used. Negative treatment responses were not used in the estimate of absolute cell counts.

Statistical Analysis

Unless otherwise noted, all statistical comparisons used two-tailed tests to determine whether there was any difference between two measures. In general, comparisons of mean values were conducted using Welch's two sample t test, and comparisons of proportions were conducted using an exact binomial test.

For Welch's two sample t test we satisfied the assumption that the data are distributed approximately normally, testing the null hypothesis that the mean of group A was equal to group B with the alternative that the means were different between the two groups.

For the exact Binomial test we satisfied the assumptions that the data are randomly sampled, the samples were independent, and the outcome measure was binary (success vs. failure). We tested the null hypothesis that the probability of success in group A was equal to the probability of success in group B, with the alternative that the probability of success differed between the two groups.

In certain comparisons (Fig. 3C,F), the Clopper-Pearson method was used to measure the

medRxiv preprint doi: https://doi.org/10.1101/2025.05.20.25328004; this version posted May 21, 2025. The copyright holder for this preprint (which was not certified by peer review) is the author/funder, who has granted medRxiv a license to display the preprint in perpetuity.
It is made available under a CC-BY 4.0 International license .

uncertainty in our estimation of proportions. The specific implementation for this method was taken from the PropCIs (v0.3-1) R package using the exactci function.

We controlled for multiple comparisons by calculating q-values using the Storey-Tibshirani procedure to account for dependency between comparisons. We controlled the False Discovery Rate (pFDR) at $\leq$ 10%. The q-value represents the probability that a given comparison is significant, given the total number of statistical comparisons made in these exploratory analyses.

Descriptive statistics were used to calculate proportions, frequencies, and mean values of variables of interest. Correlations were made using R^2 values to measure the amount of residual error relative to the error explained by the regression ((SSR – SSE) / SSR). Confidence Intervals were calculated as the mean plus or minus the critical value times the quotient of standard deviation and the square root of the number of samples (standard error). In general, the critical value was from a student t distribution on $n - 1$ degrees of freedom and $\alpha = 0.05$. Geometric means were calculated using the DescTools R package.

All statistical analyses were conducted in RStudio Version 2024.09.0 Build 375 (using R version 4.4.1). The source code for these analyses and additional documentation are available at https://github.com/P-Harvey/Poliovirus_Antigen_Specific_TCells.

Reagent List

Histopaque-1077 (Sigma #H8889)

Cell Freezing Media (Sigma #C6164)

medRxiv preprint doi: https://doi.org/10.1101/2025.05.20.25328004; this version posted May 21, 2025. The copyright holder for this preprint (which was not certified by peer review) is the author/funder, who has granted medRxiv a license to display the preprint in perpetuity.
It is made available under a CC-BY 4.0 International license .

RPMI 1640 (Cytiva #SH30096.02)

Heat-Inactivated Fetal Bovine Serum (Corning #35-011-CV)

L-Glutamine (Gibco #A2916802)

Penicillin-Streptomycin (Gibco #15070063)

Benzonase (Sigma-Aldrich #E1014-25KU)

70 µM Filters (PluriSelect #43-10070-70)

Phytohemagglutinin PHA (Roche #11249738001)

Dimethyl Sulfoxide (“DMSO”, Sigma #D8418)

Phosphate Buffered Saline (“PBS”, Corning #21-031-CV)

1% Paraformaldehyde (Alfa Aesar #43386)

UV Blue LiveDead (Invitrogen #L34962, 1:200)

Brilliant Buffer (BD Horizon #566349)

Fc Block (BioLegend #422302, 1:20)

Monocyte Block (BioLegend #426103, 1:20)

CD69 BUV661 (BD #750213, 1:100)

CD8 BUV805 (BD #612890, 1:400)

CD4 Pacific Blue (BioLegend #317424, 1:400)

CD3 BV510 (BioLegend #344828, 1:50)

CD134/OX40 BV605 (BioLegend #350028, 1:20)

CD14 BV711 (BioLegend #301838, 1:100)

CD19 BV711 (BioLegend #302246, 1:100)

CXCR5 BV750 (BioLegend #356942, 1:20)

CD279/PD-1 PE (BioLegend #329906, 1:40)

medRxiv preprint doi: https://doi.org/10.1101/2025.05.20.25328004; this version posted May 21, 2025. The copyright holder for this preprint (which was not certified by peer review) is the author/funder, who has granted medRxiv a license to display the preprint in perpetuity.
It is made available under a CC-BY 4.0 International license .

CD45 PerCP/Cy5.5 (BioLegend #368504, 1:50)

CD137 PE/Cy7 (BioLegend #309818, 1:50)

TCRgd APC (BioLegend #331212, 1:20)

a4β7 AF700 (R+D #FAB10078N, 1:40)

CD45RA BUV395 (BD OptiBuild #740315, 1:200)

CD20 BUV563 (BD OptiBuild #748456, 1:800)

CD196/CCR6 BUV661 (BD OptiBuild #750696, 1:400)

CD8 BUV805 (BD Horizon #612890, 1:100)

CD197/CCR7 BV421 (BioLegend #353208, 1:40)

CD4 PacBlue (BioLegend #317423, 1:400)

CD19 BV480 (BD #568214, 1:100)

CD3 BV510 (BioLegend #344828, 1:100)

IgM BV570 (BioLegend #314517, 1:100)

CD14 BV711 (BioLegend #301838, 1:100)

CD185/CXCR5 BV750 (BioLegend #356942, 1:100)

IgA VioBright B515 (MACS Miltenyi #130-116-886, 1:200)

MR1 5-OP-RU PE (Emory/NIH #NA, 1:100)

CD27 PE-CF594 (BD #562297, 1:100)

CD45 PerCP/Cy5.5 (BioLegend #368504, 1:40)

CD183/CXCR3 PE/Cy7 (BioLegend #353720, 1:100)

TCRgd APC (BioLegend #331211, 1:20)

a4β7 AF700 (R+D #FAB10078N, 1:40)

CD38 APC/Fire810 (BioLegend #303549, 1:100)
